# Digital health literacy and caring behaviours among Turkish hospital nurses: a cross-sectional study

**DOI:** 10.1186/s12912-026-04550-x

**Published:** 2026-04-03

**Authors:** Muzelfe Biyik, Yasemin Onal

**Affiliations:** 1 Department of Nursing Management, Faculty of Health Sciences, Kutahya Health Sciences University, Kutahya, Türkiye; 2 Afyonkarahisar Health Sciences University Training and Research Hospital, Afyonkarahisar, Türkiye

**Keywords:** Digital health literacy, Caring behaviours, Hospital nurses, Nursing practice

## Abstract

**Background:**

Digital health literacy has become an essential competence for nurses working in increasingly digitalised healthcare environments. However, limited evidence exists regarding how digital health literacy is reflected in nurses’ caring behaviours, which are central to patient-centred and quality nursing care. This study examined the relationship between nurses’ digital health literacy and their caring behaviours and determined whether digital health literacy predicted caring behaviours.

**Methods:**

This descriptive cross-sectional study was conducted with 241 nurses working in a university hospital in Türkiye, recruited using a convenience sampling method. Data were collected using a demographic information form, the Digital Health Literacy Instrument (DHLI), and the Caring Behaviours Inventory-24 (CBI-24). Descriptive statistics, Pearson correlation analysis, and multiple linear regression analysis (Stepwise method) were performed to examine associations and predictive relationships between variables.

**Results:**

Nurses reported adequate levels of digital health literacy (mean score = 2,92 ± 0,53) and high levels of caring behaviours (mean score = 5,29 ± 0,48). Digital health literacy was positively but weakly associated with caring behaviours (*r* = 0,136, *p* = 0,036). In the regression analysis, digital health training (β = 0.214, *p* = 0.003) and protection of privacy (β = 0.186, *p* = 0.012) emerged as significant predictors of caring behaviours, explaining 9.7% of the variance (R² = 0.097, F = 7.436, *p* < 0.001).

**Conclusions:**

Digital health literacy demonstrated a modest but significant contribution to caring behaviours. In particular, privacy-related competencies appear to play a critical role in linking digital literacy to caring practices. These findings highlight the need to integrate digital communication and privacy-focused competencies into nursing education and organisational training programmes to strengthen caring behaviours in digitalised healthcare settings.

## Background

Electronic health records, clinical decision support systems, telehealth applications, and mobile health technologies have now become an integral part of nurses’ daily clinical practice [1]. This rapid and unstoppable digitalization process in healthcare is fundamentally and significantly transforming nurses’ traditional approaches to direct patient care [2]. In the modern healthcare ecosystem, nurses are not only expected to demonstrate their clinical competence. In addition, they are expected to access health information in a timely manner, critically evaluate the appropriateness and reliability of this information, and integrate the data they obtain into clinical decision-making processes. At this point, digital health literacy (DHL) emerges as a fundamental competency that goes beyond the technical use of digital tools and supports the process of understanding and decision-making in clinical practice [3].

Digital health literacy is defined as the ability of individuals to research, understand, evaluate, and apply health-related information obtained from digital environments [4, 5]. From a nursing perspective, digital health literacy is a multidimensional competency that encompasses topics such as searching for digital health information, critically evaluating its reliability and appropriateness, and applying this information in clinical contexts [4, 6]. In increasingly digitized healthcare environments, nurses are often exposed to excessive information flow, which can create cognitive load in clinical decision-making processes [3]. Nurses with high levels of digital health literacy can manage this information load more effectively, reduce uncertainty, and improve clinical reasoning. This cognitive clarity can translate into caring behaviours such as providing clearer information in patient education, supporting self-care processes, and paying greater attention to privacy and security [7]. Previous studies have shown that digital health literacy is associated with individual health behaviours [8] and quality of care through nurses’ counseling roles and core nursing competencies [3, 9]. In this context, DHL can be considered not only a technical competency but also a capacity that can shape how digital information is translated into care practices in clinical settings. Studies in different countries have reported that nurses’ levels of digital health literacy are high in Jordan and Iran [7, 9], moderate in China [3], and adequate in Italy [10]. Research on this topic is limited in Türkiye, and current evidence suggests that nurses’ digital health literacy levels are generally moderate [11]. However, to our knowledge, no national study has directly examined the relationship between digital health literacy and professional caring behaviours. Previous research has shown that nurses with higher levels of digital health literacy are better able to integrate digital information into clinical decision-making processes, which positively impacts their care practices [7, 9, 10, 12]. However, the current literature has largely focused on defining nurses’ levels of digital health literacy or examining their relationship with digital attitudes and general health behaviours [8, 10, 13, 14].

Consequently, the relationship between digital health literacy and caring behaviours in routine nursing practice has not been sufficiently investigated. In the rapidly expanding digital health environment, nurses play a crucial role in the effective implementation of digital health practices in clinical settings. The World Health Organization has identified strengthening the digital competencies of health professionals as a priority for the successful integration of digital health technologies into health systems [15]. Beyond technical competence, these competencies are expected to be reflected in nurses’ daily clinical interactions and care practices. Therefore, gaining a clearer understanding of how digital health competencies relate to clinical caring behaviours is of critical importance for professional development, workforce capacity building, and health policy formulation [16]. This study seeks to address an important gap in the literature by examining the relationship between nurses’ digital health literacy and their caring behaviours in clinical practice.

This study aimed to examine the relationship between nurses’ levels of digital health literacy and their caring behaviours, as well as to identify factors associated with caring behaviours. Specifically, the study addressed the following research questions: (1) What are the levels of nurses’ digital health literacy and caring behaviours (2)? Is there a relationship between nurses’ digital health literacy and their caring behaviours (3)? Which sociodemographic characteristics and digital health literacy factors are associated with caring behaviours among nurses?

## Methods

### Design, and setting

A descriptive cross-sectional design was adopted, and data were collected using self-administered questionnaires. The study was conducted between July and November 2025 in a university hospital in Türkiye.

### Sample and participants

The study population consisted of nurses working in patient care units at the university hospital. A convenience sampling method was used to recruit participants. As participation was voluntary and based on accessibility, the possibility of selection bias cannot be excluded. Inclusion criteria were (a) actively working in patient care units and (b) willingness to participate in the study. Nurses holding managerial or administrative positions were excluded. Participation was voluntary.

A one-tailed hypothesis was specified based on prior empirical evidence consistently reporting positive associations between digital health literacy and care-related outcomes among nursing populations. The required sample size was calculated using G*Power version 3.1.9.7[17]. Based on a reported correlation coefficient of *r* = 0.193 [18], with a significance level of α = 0.05, a null hypothesis correlation of 0, and a statistical power of 80% (1 − β = 0.80), the minimum required sample size was calculated as 164.

### Data collection procedures

Data were collected through face-to-face administration within the hospital setting. Prior to data collection, ethical approval was obtained. Participants were informed about the purpose of the study, and written informed consent was obtained from all participants. Nurses who met the inclusion criteria were informed about the purpose and procedures of the study and were invited to participate voluntarily. Confidentiality was assured, and participants were informed of their right to withdraw from the study at any time without consequence. Data collection instruments included a Participant Information Form, the Digital Health Literacy Instrument (DHLI), and the Caring Behaviours Inventory-24 (CBI-24).

The Participant Information Form was developed based on a literature review and consists of 14 items evaluating the sociodemographic and professional characteristics of nurses.

### Digital health literacy instrument

Digital health literacy was assessed using the Digital Health Literacy Instrument (DHLI), developed by Van der Vaart and Drossaert [19] and adapted into Turkish by Çetin and Gümüş [20]. The tool consists of 18 items in six sub-dimensions: search, reliability, relevance, navigation, content creation, and privacy. Four sub-dimensions are rated on a 4-point Likert scale, while the navigation and privacy sub-dimensions are scored on a frequency scale using reverse coding. Total scores range from 1 to 4. Scores below 2 indicate low digital health literacy, scores between 2 and 3 indicate moderate literacy, and scores above 3 indicate high literacy. In the current study, the DHLI demonstrated strong internal consistency (Cronbach’s α = 0.921).

### Caring behaviours inventory-24

Caring behaviours were assessed using the CBI-24 scale, initially developed by Wu et al. [21] and later adapted for the Turkish population by Kurşun and Kanan [22], with its validity confirmed. The scale consists of 24 items divided into four subscales: Assurance, Knowledge-Skills, Respect, and Connectedness. Responses are recorded on a 6-point Likert scale, with higher scores reflecting stronger perceptions of caregiving behaviours. The internal consistency of the CBI-24 was high in this study (Cronbach’s α = 0.936).

### Data analysis

Descriptive statistics, including frequencies, percentages, means, standard deviations, medians, minimums, and maximums, were used to summarize the characteristics of participants and study variables. Internal consistency of the measurement instruments was assessed using Cronbach’s alpha coefficients. Normality of the data was assessed using the Shapiro-Wilk test. Since the data did not show a normal distribution, Spearman correlation analysis was performed to examine the relationships between digital health literacy and caring behaviours. Stepwise multiple linear regression analysis was performed to identify the determinants of caring behaviours. In linear regression, the assumption of normality pertains to the distribution of residuals rather than the raw independent variables [23]. Accordingly, residual normality was evaluated using skewness and kurtosis statistics, and the results indicated that the assumption of normality was satisfied. Residual normality was evaluated using skewness and kurtosis statistics (− 1 to + 1 range). Multicollinearity was examined through the Variance Inflation Factor (VIF < 5). Independence of errors was assessed using the Durbin–Watson statistic (1.834), indicating no autocorrelation. Model significance was tested using ANOVA (F = 7.436, *p* < 0.001), and the final model explained 9.7% of the variance in caring behaviours (R² = 0.097). All statistical analyses were conducted using IBM SPSS Statistics for Windows, version 27 (IBM Corp., Armonk, NY, USA).

## Results

A total of 241 nurses participated in the study. Among them, 44.8% were aged 26–34 years, 68.9% were women, 64.3% were married, and 67.2% held a university degree. In addition, 32.8% had 6–10 years of professional experience. Regarding digital use, 41.1% reported using the Internet daily for professional purposes, 58.5% considered the Internet useful for health-related decisions, and 50.6% emphasised the importance of accessing online health resources. Furthermore, 41.1% reported intermediate computer proficiency, 65.1% reported moderate digital health literacy, 81.7% had not received digital health training, and 69.3% expressed willingness to receive training on digital nursing applications (Table 1).

**Table 1:** Socio-demographic characteristics of nurses (*N* = 241)

Characteristics		*n* (%)
Age	< 25 years	26 (10.8)
	26–34 years	108 (44.8)
	35–44 years	94 (39.0)
	> 45 years	13 (5.4)
Gender	Female	166 (68.9)
	Male	75 (31.1)
Marital status	Single	86 (35.7)
	Married	155 (64.3)
Educational status Working time in profession	Associate degree	39 (16.2)
	University degree	162 (67.2)
	Postgraduate degree (master/doctorate)	40 (16.6)
	≤ 5 years	41 (17.0)
	6–10 years	79 (32.8)
	11–15 years	57 (23.7)
	> 16 years	64 (26.6)
Working in unit	Intensive care unit	77 (32.0)
	Operating theatre	26 (10.8)
	Internal medicine clinic	56 (23.2)
	Surgical clinic	51 (21.2)
	Other	31 (12.9)
Frequency of Internet searches for professional purposes	Never	49 (20.3)
	Once a month	15 (6.2)
	Once a week	51 (21.2)
	Everyday	99 (41.1)
	Several times a day	27 (11.2)
Perceived usefulness of the Internet for health decisions	Not useful at all	20 (8.3)
	Not useful	23 (9.5)
	Undecided	57 (23.7)
	Useful	141 (58.5)
Importance of accessing online health-related resources	Not useful	31 (12.9)
	Undecided	88 (36.5)
	Useful	121 (50.6)
Proficiency in using computer	Very inadequate	4 (1.7)
	Inadequate	20 (8.3)
	Intermediate	99 (41.1)
	Sufficient	79 (32.8)
	Good	39 (16.2)
Proficiency in digital health literacy	Yes	41 (17.0)
	Partially	157 (65.1)
	No	43 (17.8)
Digital health training	Yes	44 (18.3)
	No	197 (81.7)
Interest in digital nursing training	Yes	167 (69.3)
	No	74 (30.7)

The distributions of the total and subscale scores of the scales used in the study are presented in Table 2. The mean total score of the Digital Health Literacy Scale is 2.92 ± 0.53, with a median of 2.94. The mean of the total score on the Caring Behaviours Scale is 5.29 ± 0.48, and the median is 5.33 (Table 2).

**Table 2:** Descriptive statistics of the scales used in the study (*N* = 241)

	Characteristics	Min-Max	Mean ± SD (Median)
DHLI	DHLI Total	1.67-4	2.92 ± 0.53 (2.94)
	Information searching	1.33-4	2.90 ± 0.64 (3)
	Evaluating data reliability	1–4	2.69 ± 0.78 (2.67)
	Determining data relevance	1–4	2.75 ± 0.68 (3)
	Navigation skill	1–4	2.69 ± 0.81 (2.67)
	Adding content	1–4	2.98 ± 0.80 (3)
	Protecting privacy	1–4	3.52 ± 0.50 (3.67)
CBI-24	CBI-24 Total	3.58-6	5.29 ± 0.48 (5.33)
	Assurance	3.5-6	5.31 ± 0.53 (5.38)
	Knowledge-skills	3.8-6	5.56 ± 0.46 (5.8)
	Respect	3.33-6	5.11 ± 0.58 (5)
	Connectedness	3–6	5.19 ± 0.59 (5.2)

Abbreviations: CBI-24; Caring Behaviours Inventory-24; DHLI; Digital Health Literacy Instrument; SD, Standard deviation

Positive and low-level correlations were found between CBI-24 Total and Level of Interest (*r* = 0.188), Protection of Privacy (*r* = 0.142), and DHLI Total (*r* = 0.135) (Table 3).

**Table 3:** Correlation between the DHLI and the CBI-24 and sub-dimensions (*N* = 241)

Characteristics		Assurance	Knowledge-skills	Respect	Connectedness	CBI-24 Total
Information searching	r	0.150	0.047	0.062	0.076	0.090
	p	0.020*	0.464	0.340	0.240	0.163
Evaluating data reliability	r	0.109	0.114	-0.027	0.131	0.084
	p	0.090	0.076	0.678	0.042*	0.195
Determining data relevance	r	0.183	0.106	0.169	0.206	0.188
	p	0.004*	0.102	0.009*	0.001*	0.003*
Navigation skill	r	0.122	0.037	0.094	0.129	0.107
	p	0.058	0.564	0.146	0.045*	0.099
Adding content	r	0.084	0.013	-0.093	0.029	0.001
	p	0.195	0.842	0.148	0.649	0.994
Protecting privacy	r	0.139	0.158	0.005	0.203	0.142
	p	0.031*	0.014*	0.940	0.002*	0.027*
DHLI Total	r	0.179	0.102	0.038	0.172	0.135
	p	0.005*	0.114	0.557	0.007*	0.036*

* *p* < 0.05 r: Correlation coefficient

Abbreviations: DHLI; digital health literacy instrument; CBI-24: Caring Behaviours Inventory-24

The linear regression model examining factors associated with the CBI-24 total score was statistically significant (Table 4). Perceived usefulness of the Internet for health decisions was a significant negative predictor; each unit increase was associated with a 0.280-point decrease in the CBI-24 score (β = −0.280, 95% CI: −0.229, − 0.052; *p* = 0.002). Computer proficiency was positively associated with caring behaviours, where each unit increase resulted in a 0.196-point increase in the CBI-24 score (β = 0.196, 95% CI: 0.023, 0.182; *p* = 0.012). Additionally, nurses who had received digital health training scored 0.189 points higher than those who had not (β = 0.189, 95% CI: 0.063, 0.405; *p* = 0.008). Similarly, proficiency in digital health literacy was also identified as a significant positive predictor of caring behaviours (β = 0.183, 95% CI: 0.026, 0.341; *p* = 0.022). Other variables—including age, gender, marital status, education level, years in the profession, years in the unit, frequency of Internet searches, importance of accessing online health resources, willingness to receive digital nursing training, and the DHLI subdimensions (search, relevance, navigation, content, privacy) as well as the DHLI total score—did not show statistically significant associations with CBI-24 total scores. Multicollinearity diagnostics indicated acceptable Variance Inflation Factor (VIF) values for all predictors (Table 4).

**Table 4:** Linear regression analysis for the caring behaviours scale (*N* = 241)

Characteristics	β	S.H.	St. β	t	*p*	95% CI (Lower)	95% CI (Upper)	VIF
Constant	4.455	0.217		20.539	< 0.001*	4.027	4.882	
Protecting privacy	0.207	0.060	0.216	3.463	0.001*	0.089	0.325	1.036
Digital health training	0.223	0.078	0.180	2.844	0.005*	0.068	0.377	1.060
Educational status	0.199	0.079	0.155	2.527	0.012*	0.044	0.355	1.001
Proficiency in digital health literacy	0.183	0.080	0.144	2.299	0.022*	0.026	0.341	1.043
F:7.436, *p* < 0.001*				R^2^ : 0.112, Adjusted R^2^ : 0.097				

## Discussion

To our knowledge, limited research has examined the relationship between nurses’ digital health literacy and their caring behaviours. This pattern suggests that digital competence alone may be insufficient to influence relational dimensions of care unless supported by organisational and contextual conditions. The generally adequate levels of digital health literacy and the high levels of caring behaviours observed in this sample may reflect nurses’ adaptation to digital technologies within the specific institutional context of this study. Specifically, the dimension of privacy protection showed the strongest correlation with caring behaviours, highlighting that respect for patient privacy remains a fundamental component of nursing care in digital environments. Furthermore, the relationships between receiving digital health education, perceived digital competence, and caring behaviours suggest that digital skills can enhance the visibility and effectiveness of nurses’ roles within the care process.

This study found that nurses’ digital health literacy levels were at the desired level. Studies in Jordan and Iran reported that digital health literacy among nurses was at the desired level [7, 9], while in China it was at a moderate level [3] and in Italy at an adequate level [10]. These findings demonstrate that the results of the current study are consistent with international evidence and suggest that nurses’ digital health literacy is generally at an acceptable level, but could be further improved. Within the context of the Turkish healthcare system, the national digital health transformation strategy and the widespread adoption of electronic health records may have contributed to nurses being exposed to digital tools more frequently in their daily practice [24]. However, unlike in some OECD countries, digital literacy has not yet been systematically integrated into nursing education and professional development processes [25]. Furthermore, digital transformation requires not only technical infrastructure but also ethical sensitivity and a culture of continuous learning [26]. In this context, exposure to digital systems in Türkiye may increase technical knowledge, but the lack of structured education may limit how digital competencies are reflected in the relational and ethical dimensions of care. Therefore, digital health literacy competencies can shape observable caring behaviours. Digital health literacy is identified as a priority area for Universal Health Coverage in the World Health Organization’s Regional Digital Health Action Plan for the European Region (2023–2030). The Action Plan emphasizes that all health professionals must be equipped to use digital health tools “to cover health information for other health professionals and share this information with them” [15]. This global perspective does not limit digital health literacy to a technical competency but is consistent with the need to conceptualize it as an ethical and relational capacity integrated into health systems.

**Fig. 1:**
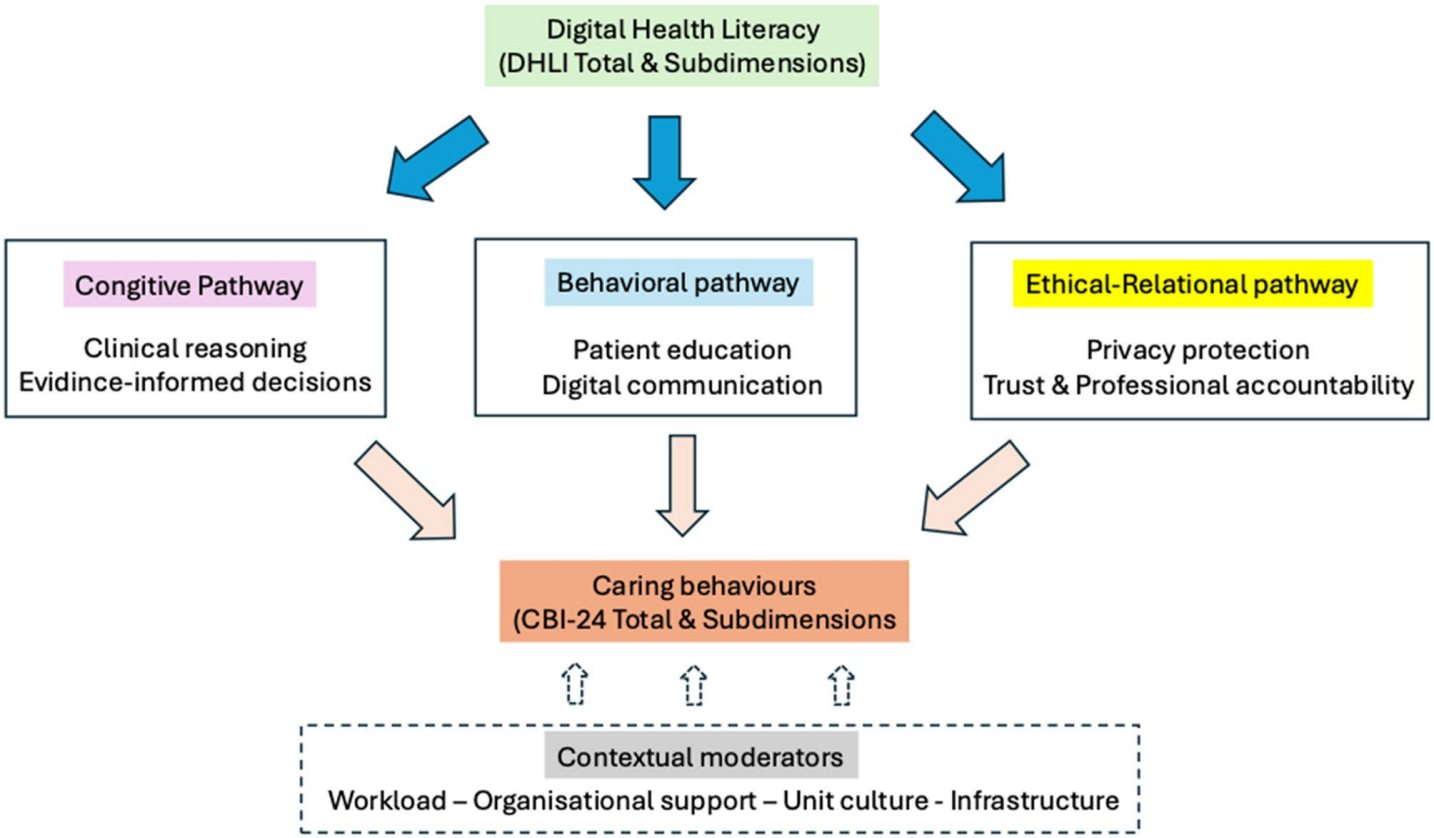
Conceptual framework: the mechanism of digital health literacy on caring behaviors

From this perspective, digital health literacy can be defined as a concept that influences caring behaviours through several interrelated pathways. First, through a cognitive pathway, it can enhance information evaluation and relevance determination, clinical reasoning, and evidence-based decision-making processes. Second, through a behavioural pathway, it can facilitate information exchange and support patient engagement through digital communication skills and patient education practices. Third, through an ethical-relational pathway, it can strengthen privacy protection, data management trust, and professional accountability. This conceptual framework demonstrates that digital competencies shape caring behaviours through cognitive, communicative, and ethical processes. However, digital health literacy acts as a facilitating mechanism for this effect, depending on organizational and contextual conditions. The proposed conceptual framework is shown in Fig. 1.

This study revealed that sub-dimensions of digital health literacy related to assessing reliability and navigation skills scored lower than other dimensions. Assessing reliability refers to the ability to critically evaluate the accuracy and validity of online health information [19]. While studies in Jordan and Iran reported higher levels in this dimension, they similarly highlight the need for training focused on critically evaluating online information [7, 9]. The findings of this study suggest that nurses may experience difficulties in confidently evaluating and discriminating information they encounter in digital environments in a clinical context. The rapidly changing, dense, and sometimes contradictory nature of digital health information can create uncertainty and distrust, hindering the integration of information into clinical decision-making processes [3, 27]. Lower scores in reliability appraisal may indicate epistemic vulnerability in digital environments, particularly under conditions of high information density. Similarly, navigation skills, defined as the ability to systematically and effectively access health information on digital platforms, were among the sub-dimensions that received relatively lower scores in this study [19]. In contrast, studies in Jordan and Iran reported higher levels of navigation skills [7, 9]. This discrepancy may be explained by nurses having limited experience in searching digital information for clinical purposes, difficulties related to information overload, or the user-friendliness of institutional digital systems [3]. These findings may indicate that the effective use of digital information in the care process could be influenced not only by individual competencies but also by contextual factors such as digital infrastructure and experiential learning opportunities within the work environment.

In this study, the highest average score among the subdimensions of digital health literacy was obtained in the dimension of privacy protection. This result indicates that nurses may have a high level of awareness regarding their ethical responsibilities concerning patient information in digital environments. Privacy in the digital environment is not only a technical data security issue; it also reflects the continuation of the trust relationship between nurses and patients in the digital context [6]. The important relationship between privacy protection and caring behaviours shows that digital competencies can influence care practices through ethical and relational means. Protecting patient information in the digital environment has been defined as a fundamental professional competency that directly contributes to the quality of care [7, 9]. This result may indicate a patient-centered approach where the patient is not merely positioned as data but recognized as a person whose dignity must be protected. The emergence of privacy protection as the strongest determinant of caring behaviours suggests that ethical awareness in digitalized healthcare may be closely linked to trust, professional responsibility, and humane care. This can be interpreted as part of the nurse’s digital ethical role and strengthens accountability and respect for patient rights [14]. Evidence in the literature regarding privacy protection is mixed; some studies report low levels of this competency [9], while others show adequate levels [7]. The relatively high score for the privacy sub-dimension in the Turkish adaptation study [20] suggests that cultural sensitivities regarding patient privacy and existing legal regulations in Türkiye may further reinforce this awareness [28].

This study has revealed a positive but weak correlation between digital health literacy and caring behaviours. Although the effect size is low (*r* = 0.136), this result should not be interpreted as indicating that digital health literacy is clinically insignificant. In complex clinical settings, the relationship between digital health literacy and caring behaviours involves numerous interacting variables and is multidimensional and context-sensitive. The literature indicates that heavy workloads, staff shortages, and time pressure may limit nurses’ capacity to translate digital information into concrete care actions [8, 14]. Similarly, inadequate technological infrastructure and limited management support may hinder the effective integration of digital competencies into clinical routines [6, 14]. Psychological factors such as information anxiety have also been reported to hinder the safe use of digital information in clinical settings [3]. Therefore, the weak correlation observed in this study may reflect that DHL is conditionally activated in specific organizational environments rather than the absence of a meaningful concept.

The fact that digital health literacy explains only 9.7% of the variance indicates that caring behaviours are shaped by broader systemic determinants. The unexplained 90.3% variance may reflect structural, interpersonal, and organizational effects that were not directly measured in this study. Previous research indicates that caring behaviours are strongly influenced by leadership style, organizational climate, team culture, workload distribution, and staff competence [6, 14]. High workload and time constraints may reduce opportunities for relational interactions, independent of individual competence [8, 14]. Similarly, inadequate organizational support and limited technological infrastructure may constrain the translation of professional competencies into observable practice behaviours [6, 14, 27]. These findings suggest that care services should be understood as a result of organizational conditions. Therefore, incorporating organizational structure, workload, and contextual variables into future research models may contribute to a better understanding of how digital health literacy interacts with the implementation environment to influence caring behaviours.

The positive relationship between digital health education, graduate education level, and caring behaviours indicates that structured and goal-oriented educational practices can strengthen nurses’ confidence in using digital tools. Research indicates that digital health literacy increases with education level and contributes to nurses managing their information load more systematically and transferring evidence-based information into care practices [3, 10, 14]. Alipour and Payandeh [7] stated that digital health education increases digital health literacy. Ahmed et al. [29] found that healthcare workers with high computer literacy had higher levels of digital health literacy. In the current study, the fact that a significant proportion of nurses requested training despite not having previously received training in this area indicates a clear need for the development of digital capacity. This highlights the importance of integrating it into nursing curricula, in-service training programs, and institutional policies with a holistic approach [14, 30]. Clinical training may aim to develop competencies such as evaluating the reliability of online information, providing information to patients in a clear and understandable manner, and protecting confidentiality. Scenario-based simulations and case-based learning activities can demonstrate how digital competencies can be translated into caring behaviours such as improving patient understanding, supporting self-care, and building trust in digital interactions [14, 31]. However, strategies such as management support, intergenerational learning models (e.g., reverse mentoring), and human-centered remote counseling skills can also facilitate the integration of digital competency into daily clinical practice [6].

### Implications for practice

The findings indicate that digital health literacy contributes to caregiving behaviours through the dimension of privacy and confidentiality. Initiatives aimed at enhancing nurses’ digital health literacy should prioritize protecting privacy, critically evaluating digital information, and developing patient-centered digital communication skills. Integrating digital literacy content into undergraduate curricula and structured in-service training programs can support nurses in transforming their digital competencies into safe, ethical, and humane care practices.

### Limitations

In this study, caregiving behaviours were measured through self-report, and the effect of social desirability bias should not be overlooked. In addition, the use of convenience sampling may limit the representativeness of the sample, and the possibility of selection bias should be considered. The relatively limited variance explained by the regression model indicates that caregiving behaviours are multidimensional and cannot be explained solely by digital health literacy. Contextual variables such as organizational support, workload, and workplace characteristics were not included in the model, creating a limitation in the interpretation of the findings. Considering these variables in future studies may contribute to a more comprehensive understanding of the relationships. Furthermore, longitudinal or intervention-based research designs would be more explanatory in revealing the direction and continuity of the observed relationships.

## Conclusion

This study demonstrates that nurses in Türkiye possess the desired level of digital health literacy and high-level caring behaviours. However, a positive but weak correlation was found between digital health literacy and caring behaviours. This indicates that digital health literacy alone does not explain caring behaviours and that it has a multi-layered and context-sensitive structure. The emergence of the privacy dimension as the strongest sub-dimension highlights the role of ethical sensitivity in digital care. The correlation between variables such as receiving digital health education, having a higher level of education, and having a high perception of digital competence and caring behaviours points to the importance of education. To this end, educational initiatives aimed at developing digital health literacy should adopt a holistic care approach alongside technical skills, strengthen ethical awareness regarding patient privacy, and promote the secure use of digital information.

## Data Availability

The datasets generated and/or analysed during the current study are available from the corresponding author on reasonable request.
